# Discovery and Structural Characterization of a Highly Protective Neutralizing Antibody Targeting the Mpox Virus A35R Protein

**DOI:** 10.1002/mco2.70804

**Published:** 2026-06-23

**Authors:** Shimeng Bai, Shuo Song, Xin Wang, Yuxin Xiao, Yun Long, Fenfang Wu, Fuxiang Wang, Zhongyi Fan, Jianqing Xu, Maozhou He, Hongzhou Lu

**Affiliations:** ^1^ Biotherapy Clinical Research Center Shenzhen Key Laboratory of Pathogen and Immunity National Clinical Research Center for Infectious Disease Shenzhen Third People's Hospital The Second Affiliated Hospital Southern University of Science and Technology Shenzhen P. R. China; ^2^ Faculty of Life and Health Sciences Shenzhen University of Advanced Technology Shenzhen P. R. China; ^3^ School of Pharmaceutical Science and Technology Hangzhou Institute for Advanced Study University of Chinese Academy of Sciences Hangzhou P. R. China; ^4^ Clinical Center of Biotherapy Zhongshan Hospital & Institutes of Biomedical Sciences Fudan University Shanghai P. R. China

**Keywords:** A35R, Cryo‐EM structure, mpox virus (MPXV), mRNA‐LNP, neutralizing antibody, therapeutic protection

## Abstract

The recent resurgence of the mpox virus (MPXV) has raised global health concerns due to its potential to cause severe illness in vulnerable populations. Targeting the extracellular enveloped virus (EEV) protein A35R is a promising strategy to restrict viral dissemination within the host. In this study, we combined mRNA‐LNP immunization with the Beacon Optofluidic system to rapidly screen and isolate high‐affinity monoclonal antibodies. One of these antibodies, 17H1, exhibited exceptional neutralization against authentic MPXV. Cryo‐electron microscopy (Cryo‐EM) analysis revealed that 17H1 binds to a specific epitope at the distal ends of the A35R dimer. The interaction is stabilized by a unique network of hydrogen bonds and salt bridges, particularly involving residue E120, distinguishing its binding mode from previously reported A35R antibodies. Furthermore, 17H1 exhibited complete protective efficacy against MPXV infection in vivo and significantly reduced pulmonary viral load and lung pathogenesis. These findings highlight 17H1 as a promising therapeutic candidate for novel mpox interventions.

## Graphical abstract

High‐affinity monoclonal antibodies (mAbs) targeting the MPXV EEV protein A35R were rapidly isolated using mRNA‐LNP vaccination and the Beacon single‐cell sorting Optofluidic platform, followed by functional profiling. One mAb, 17H1, potently neutralized authentic MPXV and conferred complete protection in vivo. Cryo‐EM revealed binding to a novel conformation‐dependent epitope at the distal ends of the A35R dimer.

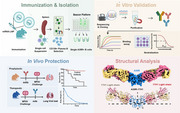

## 1 Introduction

Mpox (formerly monkeypox) is an infectious zoonotic disease caused by the mpox virus (MPXV), an orthopoxvirus in the Poxviridae family. MPXV Clade IIb caused the 2022 global epidemic, and a new round outbreak of MPXV Clade Ib occurred in 2024, prompting the WHO to declare mpox a Public Health Emergency of International Concern (PHEIC) twice in 2 years [1]. As of November 30, 2025, 142 countries and regions reported 175,415 confirmed cases and 467 deaths [2].

Currently, few antiviral drugs have been available for the treatment of mpox. ACAM2000, JYNNEOS (MVA‐BN), and LC16m8 vaccines are currently available for the prevention of MPXV infections, although they may not be suitable for vulnerable populations due to serious adverse effects or safety concerns [3, 4, 5, 6]. Tecovirimat, an approved antiviral drug used for orthopoxviruses, has also shown limited ability to speed recovery in patients infected with MPXV Clade I strains [7]. Thus, developing effective treatments against the MPXV and its variants is still urgently needed.

Monoclonal antibodies (mAbs) could be a useful way to control infection [8, 9, 10], but so far, no MPXV‐specific mAb has entered clinical trials. Orthopoxviruses produce two infectious forms: intracellular mature virus (IMV) and extracellular enveloped virus (EEV) [11]. In general, proteins in IMV mainly block cell entry and membrane fusion, while EEV proteins play a critical role in cell‐to‐cell spread and host infection [12, 13, 14, 15]. Research in vaccinia virus (VACV) suggests that antibodies against EEV proteins such as B5 and A33 can protect mice against severe outcomes [16, 17, 18, 19]. Remarkably, immunological studies have shown that individuals with historic smallpox vaccination can maintain strong A35R‐binding antibodies for over 40 years, suggesting that A35R may be a promising target for inducing long‐lasting immune memory [20, 21]. However, though MPXV A35R is highly similar to VACV A33R, small sequence differences can change whether antibodies cross‐react. For example, the mAb 1G10 could strongly bind to VACV A33R but does not identify MPXV A35R [19, 22, 23]. Thus, elucidating the structural basis and precise epitopes of A35R is essential to understanding which regions confer protection and how antibodies neutralize MPXV across different lineages.

In this study, we combined rapid mRNA‐LNP immunization with an optofluidic system‐based Beacon high‐throughput single‐cell sorting method to obtain high‐affinity antibodies directly from enriched spleen plasma cells, as we previously established [24]. Immunization with 5 µg A35R‐Fc mRNA‐LNP, we isolated and characterized five neutralizing antibodies. We test their binding, neutralization, and in vivo protective efficacy. We further elucidated the cryo‐EM structure of key A35R domains and mapped the epitope of a specific antibody, 17H1. Together, these data delineate the protective potential of A35R‐targeting antibodies and support the development of antibody‐based interventions for mpox.

## 2 Results

### 2.1 Isolation of MPXV‐specific A35R Antibodies

**FIGURE 1:**
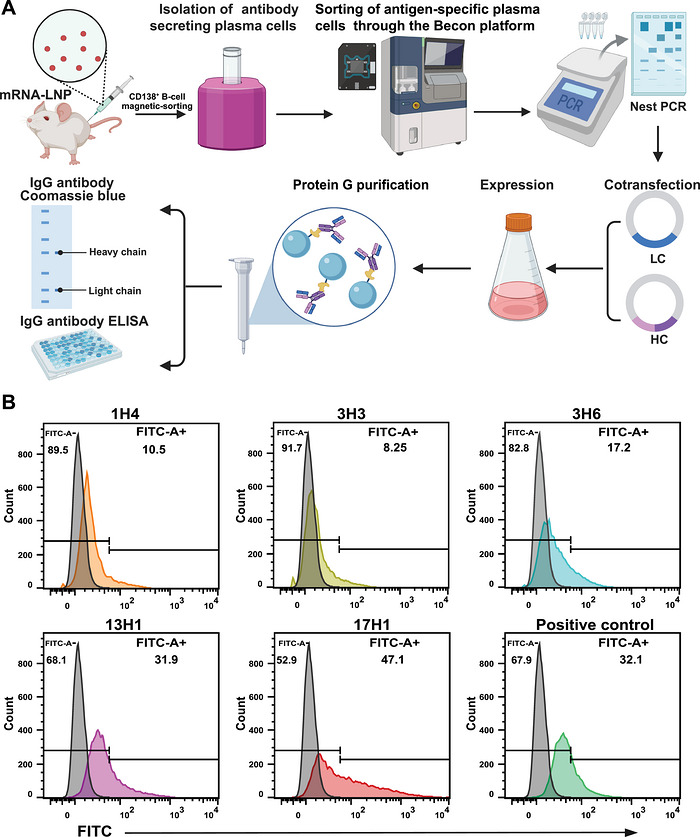
Generation of mAbs targeting MPXV A35R from mRNA‐LNP‐immunized mice. (A) Schematic of the Beacon platform‐based high‐throughput screening for isolation of A35R‐specific monoclonal antibodies (mAbs) from mRNA‐LNP vaccinated mice. The illustration was created using BioRender. (B) The abilities of five mAbs binding to the surface of A35R protein‐expressing HEK293T cells were examined by flow cytometry, with a commercial monoclonal antibody as the positive control.

Our prior finding indicated that the A35R‐Fc chimeric protein could enhance the affinity and immunogenicity of the MPXV A35R protein [25]. Specifically, the Fc domain enhances affinity through dimerization and improves immunogenicity through Fc receptor‐mediated antigen presentation. To address the poor immunogenicity without adjuvants or aggregation limitations of recombinant proteins, and further expand anti‐mpox prevention strategies, BALB/c mice were immunized intramuscularly with 5 µg A35R‐Fc mRNA‐LNP at a 3‐week interval (Figure 1A). Then, we measured the serum antibody titers 10 days after booster immunization. The mice with antibody titers exceeding 10^6^ were selected for further cell sorting.

Next, the selected spleens were harvested 5 days after the third injection to prepare a single‐cell suspension. Pan B cells were isolated using a negative selection method according to the MojoSort Mouse B Cell Isolation Kit protocol. Following, antibody‐secreting cells (ASCs) were enriched by positive selection for CD138+ cells using the EasySep Mouse CD138+ Kit. Then, using an optofluidic system‐based Beacon high‐throughput single‐cell sorting method [24], we exported A35R antigen‐reactive B cells from the instrument. DNA sequencing revealed the heavy‐chain variable region (VH) and light‐chain variable region of individual positive single B cells. Based on sequence similarity, we identified five VH‐VL paired sequences (1H4, 3H3, 3H6, 13H1, and 17H1). It showed that the heavy chains of all five antibodies were derived from the IGHV1 germline gene, whereas their light chains were IGKV6 for 1H4, IGKV3 for 13H1, and IGKV14 for 3H3, 3H6, and 17H1 (Table S1). In addition, the somatic hypermutation frequencies in the heavy chains were 3.5% (1H4), 3.8% (3H3), 4.4% (3H6), 2.4% (13H1), and 2.7% (17H1), while those in the light chains were 2.1% (1H4), 1.1% (3H3), 1.1% (3H6), 0.3% (13H1), and 1.4% (17H1) (Table S1). The five monoclonal antibodies had heavy‐chain complementarity‐determining region 3 (HCDR3) loops of 8–19 amino acids and κ‐chain CDR3 (KCDR3) loops of 9 amino acids (Table S2). To further isolate effective neutralizing antibodies and expand their clinical application potential, we replaced their constant regions with a human immunoglobulin G1 (IgG1) backbone and synthesized these antibody gene sequences, then co‐transfected heavy‐ and light‐chain plasmids into HEK293F cells. MAbs were obtained from the supernatant and purified using Protein A after 5 days (Figure S1).

To further verify the specific recognition of the MPXV A35R protein by the five antibodies, we expressed full‐length A35R on the surface of HEK293T cells. Flow cytometry analysis showed that the 1H4, 3H3, and 3H6 showed relatively low binding rates to A35R‐expressing HEK293T cells, with 10.5%, 8.25%, and 17.2%, respectively; while 13H1 exhibited a binding rate comparable to that of a commercial positive‐control monoclonal antibody (31.9% and 32.1%, respectively), whereas 17H1 bound markedly better than the other antibodies (Figure 1B). These results indicate that all five antibodies bind A35R specifically.

### 2.2 Characterization of MPXV A35R‐Specific Antibodies

**FIGURE 2:**
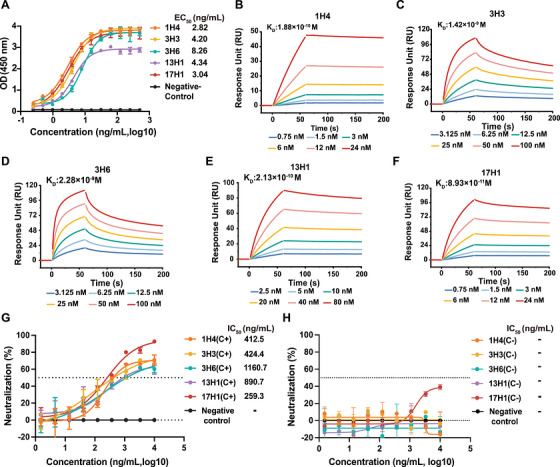
Affinity and neutralization activity of mAbs against MPXV. (A) Binding of five isolated mAbs (1H4, 3H3, 3H6, 13H1, 17H1) to the MPXV A35R protein as assessed by enzyme‐linked immunosorbent assay (ELISA). (B–F) Representative binding affinity of the isolated five antibodies to the A35R protein as determined by surface plasmon resonance (SPR). (G and H) Neutralization of the five mAbs against authentic MPXV measured by focus reduction neutralization test (FRNT) with (G) or without (H) complement. Data are presented as the mean values of two experiments with standard deviation.

To better understand the binding and neutralizing capabilities of the five A35R monoclonal antibodies, we first performed ELISA and surface plasmon resonance (SPR) assays to determine their binding kinetics. It showed all five antibodies bound strongly to the A35R protein, with EC_50_ values ranging from 2.82 to 8.26 ng/mL (Figure 2A). SPR affinity results indicated that 17H1 exhibited extremely high affinity for the A35R protein, with a K_D_ value of 0.09 nM (Figure 2F, Table S3), while the other four antibodies showed lower affinity for A35R, with K_D_ values of 0.21 nM (1H4), 1.60 nM (3H3), 2.47 nM (3H6), and 0.42 nM (13H1) (Figure 2B–E, Table S3).

Next, we evaluated their neutralizing activity against the authentic MPXV virus using a focus reduction neutralization test (FRNT). The findings demonstrated that all five antibodies exhibited neutralizing efficacy against MPXV, with 17H1 exhibiting the highest neutralization activity, characterized by a half‐maximal inhibitory concentration (IC_50_) of 259.3 ng/mL in the presence of complement, which was lower than that of the other isolated antibodies, including 1H4 (412.5 ng/mL), 3H3 (424.4 ng/mL), 3H6 (1160.7 ng/mL), and 13H1 (890.7 ng/mL) (Figure 2G). In contrast, the neutralizing efficacy of the five monoclonal antibodies was significantly reduced in the absence of complement; none of them showed neutralizing activity even at a concentration of 10 µg/mL (Figure 2H), which is consistent with the previously confirmed complement dependence of neutralizing antibodies targeting EEV [15, 16, 17, 18, 19]. To better assess the binding affinity of 17H1, we also performed another experiment using A27D7, a previously described murine monoclonal antibody with potent cross‐neutralizing [18]. Competitive SPR assays showed that 17H1 competes with A27D7 for binding to the A35R protein, indicating that the epitope recognized by 17H1 partially overlaps with the epitope recognized by A27D7 (Figure S2A). Furthermore, the binding affinity of 17H1 was 0.09 nM, which is higher than that of A27D7 (2.31 nM) (Figure S2B). These findings suggest that 17H1 is a high‐affinity A35R antibody with superior neutralizing potency against MPXV and should therefore be prioritized for further development.

### 2.3 Protection Efficacy Evaluation of 17H1 against Lethal MPXV Challenge

**FIGURE 3:**
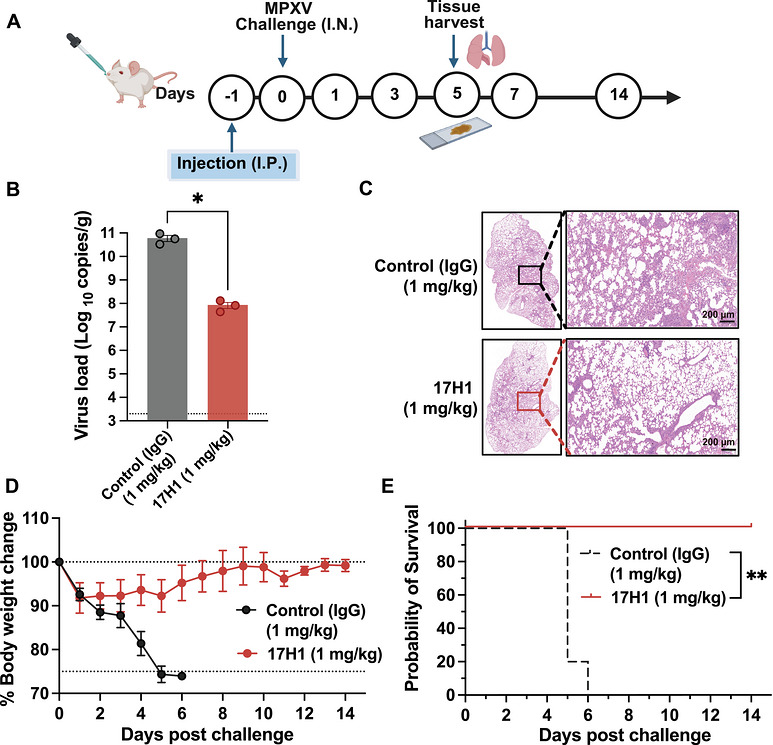
**P**rophylactic efficacy of 17H1 against lethal MPXV infection in mice. Female 6‐ to 8‐week‐old BALB/c mice were challenged intranasally (i.n.) with MPXV (8 mice per group, 1 × 10^6^ FFU dose/mouse in 50 µL). (A) Schematic overview of the assessment of the prophylactic efficacy of 17H1. BALB/c mice were administered 1 mg/kg via intraperitoneal (i.p.) injection 24 h before infection; the control ones received an equal isotype IgG. (B and C) The lung tissues (three mice from each group) were collected 5 days post‐infection from prophylactic groups, and virus loads were determined by using quantitative real‐time PCR (B). The dotted line indicates the limit of detection. Histopathological analysis of the lung tissues from the prophylactic groups was performed, and representative histological changes are shown (Scale bars are labeled in the figures; scale bar = 200 µm) (C). (D) Weight loss of BALB/c mice following MPXV challenge in prophylactic groups. Weight changes in all groups were monitored for 14 days post‐infection; a weight loss exceeding 25% was considered death. (E) Survival curves of BALB/c mice receiving 17H1 prophylactically versus control (IgG) groups. The data were presented as the mean ± standard deviation. For survival data in (E), statistical significance was assessed using the Mantel–Cox log‐rank test. (∗*p* < 0.05, ∗∗*p* < 0.01).

Neutralizing antibodies can provide passive protection against viral infection via prophylactic and therapeutic mechanisms. To evaluate the prophylactic effect of 17H1 in vivo, BALB/c mice were inoculated intranasally with a lethal dose of MPXV, and then administered 17H1 via intraperitoneal (i.p.) injection 24 h before infection (1 mg/kg; Figure 3A). Control mice received an isotype IgG. Mice pretreated with 17H1 showed complete protection, with 100% survival during the 14‐day observation period (Figure 3E). In contrast, all IgG‐treated control mice succumbed to infection by day 6, coinciding with body‐weight loss exceeding 25% (Figure 3D). To quantify viral replication, lungs were collected at Day 5 post‐infection for viral load analysis. 17H1 (7.94 log_10_) pretreatment significantly reduced viral DNA levels compared with the IgG group (10.79 log_10_) (*p* < 0.05; Figure 3B). Consistently, histopathological analysis revealed markedly reduced lung lesions in the 17H1 prophylaxis group relative to controls (Figure 3C).

**FIGURE 4:**
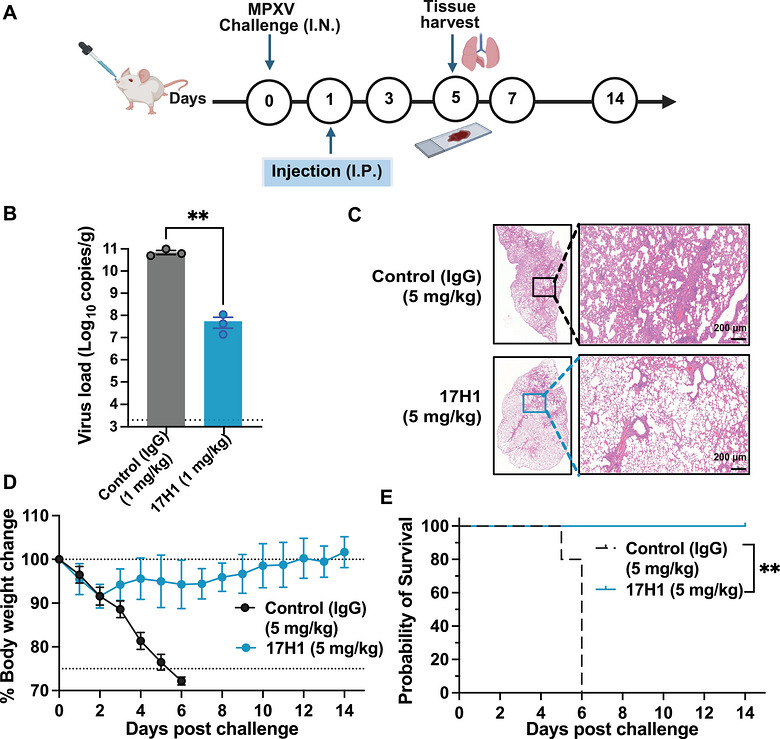
Therapeutic efficacy of 17H1 against lethal MPXV infection in mice. Female 6–8‐week‐old BALB/c mice were challenged intranasally (i.n.) with MPXV (8 mice per group; 1×10^6^ FFU per mouse in 50 µL). (A) Experimental design for evaluating the therapy efficacy of 17H1 in BALB/c mice. The mice were treated with 5 mg/kg by i.p. injection 24 h after infection, and the equal isotype IgG was used as a control. (B) Viral loads in lung tissues collected at 5 days post‐infection (d.p.i.) from therapy groups (three mice per group) were analyzed by quantitative real‐time PCR. The dash line indicates the limit of detection. (C) Histopathological analysis of lung tissues from therapy groups at 5 d.p.i. Representative H&E‐stained lung sections are shown. Scale bars are indicated in the images (200 µm). (D) Body weight changes following MPXV challenge in therapy groups. Body weight was monitored daily for 14 days post‐infection (d.p.i.); mice with over 25% weight loss were considered dead and removed from subsequent monitoring. (E) Survival curves of mice receiving 17H1 therapy versus control (IgG). Data are presented as mean ± standard deviation. Statistical significance was determined using one‐way ANOVA for viral load comparisons and the Mantel–Cox log‐rank test for survival. (∗*p* < 0.05, ∗∗*p* < 0.01).

To further assess the therapeutic potential of 17H1, BALB/c mice were intranasally (i.n.) challenged with a lethal dose of MPXV and treated with 17H1 by i.p. injection 24 h after infection (5 mg/kg; Figure 4A), with an isotype IgG as control.

Therapeutic administration of 17H1 also provided complete protection, with 100% survival and sustained body weight over the 14‐day period (Figure 4E). In contrast, IgG‐treated mice exhibited rapid clinical decline, and all reached the humane endpoint by day 6, when body‐weight loss exceeded 25% (Figure 4D). Lung viral load measured at Day 5 post‐infection showed that 17H1 treatment (7.75log_10_) reduced viral DNA by over 1000‐fold compared with the controls (10.85 log_10_) (*p* < 0.01; Figure 4B). Histopathological examination further confirmed substantially alleviated lung tissue damage in the 17H1‐treated group (Figure 4C).

Together, these results demonstrate that 17H1 provides robust prophylactic and therapeutic protection against lethal MPXV infection, significantly suppressing viral replication and lung pathology in vivo.

### 2.4 Cryo‐EM Analysis of the MPXV A35R Protein in Complex With 17H1

**FIGURE 5:**
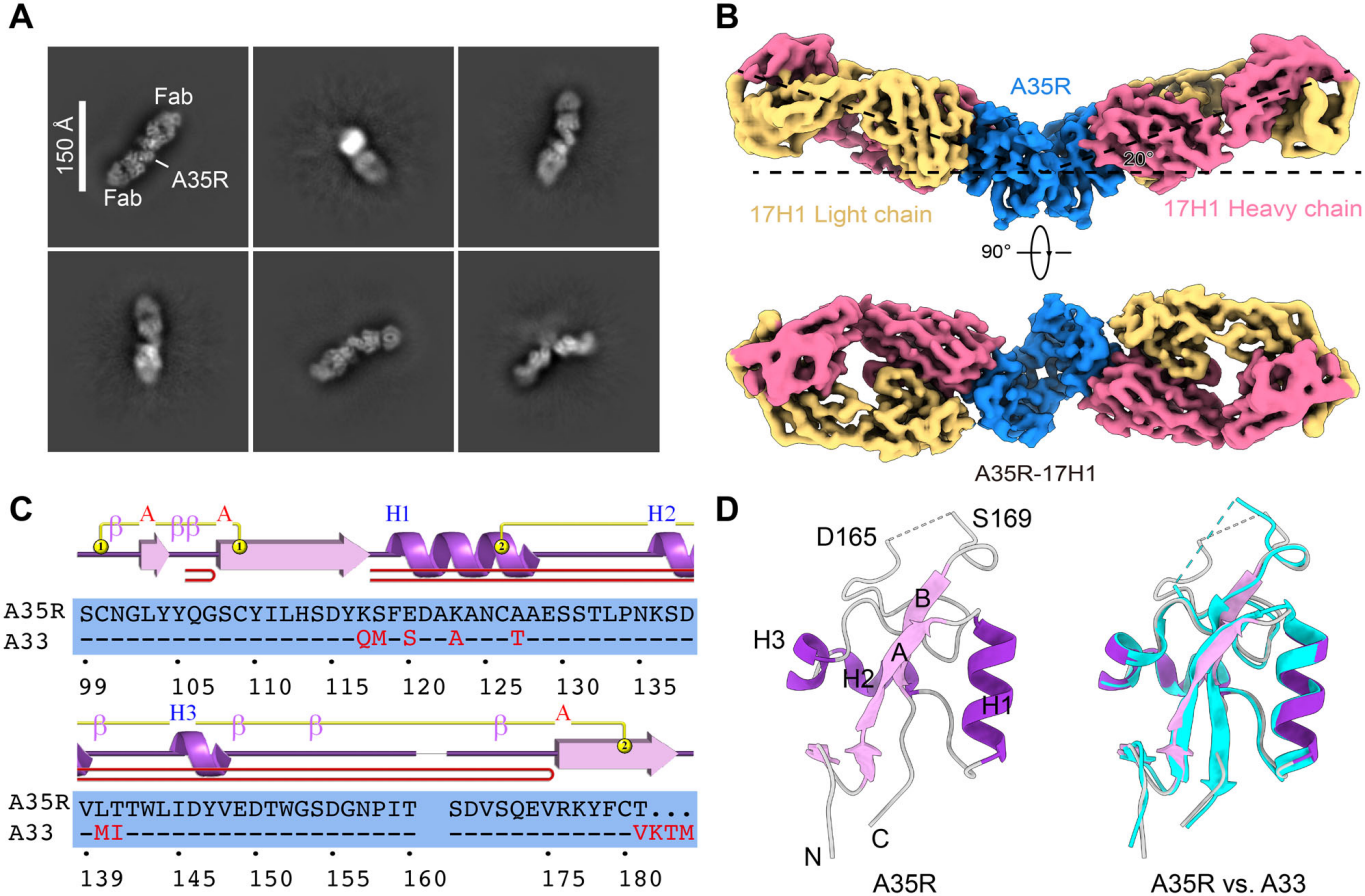
Cryo‐EM structure of MPXV A35R in complex with the 17H1 Fab. (A) Representative two‐dimensional (2D) class averages of the MPXV A35R‐17H1 complex. (B) Cryo‐electron microscopy (Cryo‐EM) structure of the MPXV A35R‐17H1 Fab complex. The density map is shown as a surface and colored according to the fitted atomic model, with MPXV A35R in blue, the 17H1 heavy chain in pink, and the light chain in yellow. (C) Secondary structure assignment of the MPXV A35R protein sequence and sequence alignment with VACV A33. β‐strands (B1‐B3) and α‐helices (H1‐H3) are indicated. (D) Ribbon representation of the A35R monomer, colored and labeled to correspond to the topology diagram (left). Structural superposition of MPXV A35R with VACV A33 is shown on the right, with A33 colored in cyan.

To elucidate the molecular mechanism by which the antibody 17H1 targets the MPXV membrane protein A35R, we prepared a complex of the ectodomain of A35R (a.a. 57‐181) bound to the Fab fragment of 17H1 (hereafter referred to as A35R–17H1). Gel filtration and SDS‐PAGE analysis confirmed that A35R forms a stable complex with the Fab, eluting at a retention volume of approximately 12 mL (Figure S3A). The complex was concentrated to ∼ 0.4 mg/mL for single‐particle cryo‐EM analysis (Figure S3B). Two‐dimensional (2D) classification indicated that A35R adopts a dimeric conformation, with each dimer bound by two Fab fragments of the 17H1 (Figure 5A,B). Following refinement, we obtained a cryo‐EM map with 3.63 Å resolution (Figure S3C,D; Table S4). Local resolution at the binding interface reached close to 3 Å, enabling near‐complete model building of the complex (Figure S4).

In the structure, only residues 99–165 and 169–181 of A35R are well resolved, likely due to the flexibility of residues 57–98 and 166–168, a feature reminiscent of the corresponding region in VACV A33 (Figure 5C). Structural analysis further reveals that A35R shares a conserved architecture with A33, consisting of three α‐helices (H1–H3) and three β‐strands (B1–B3) (Figure 5C,D). Superposition of A35R and A33 demonstrates extensive structural similarity, with a Cα root‐mean‐square deviation (RMSD) of 0.7 Å over residues 99–181 (Figure 5D).

**FIGURE 6:**
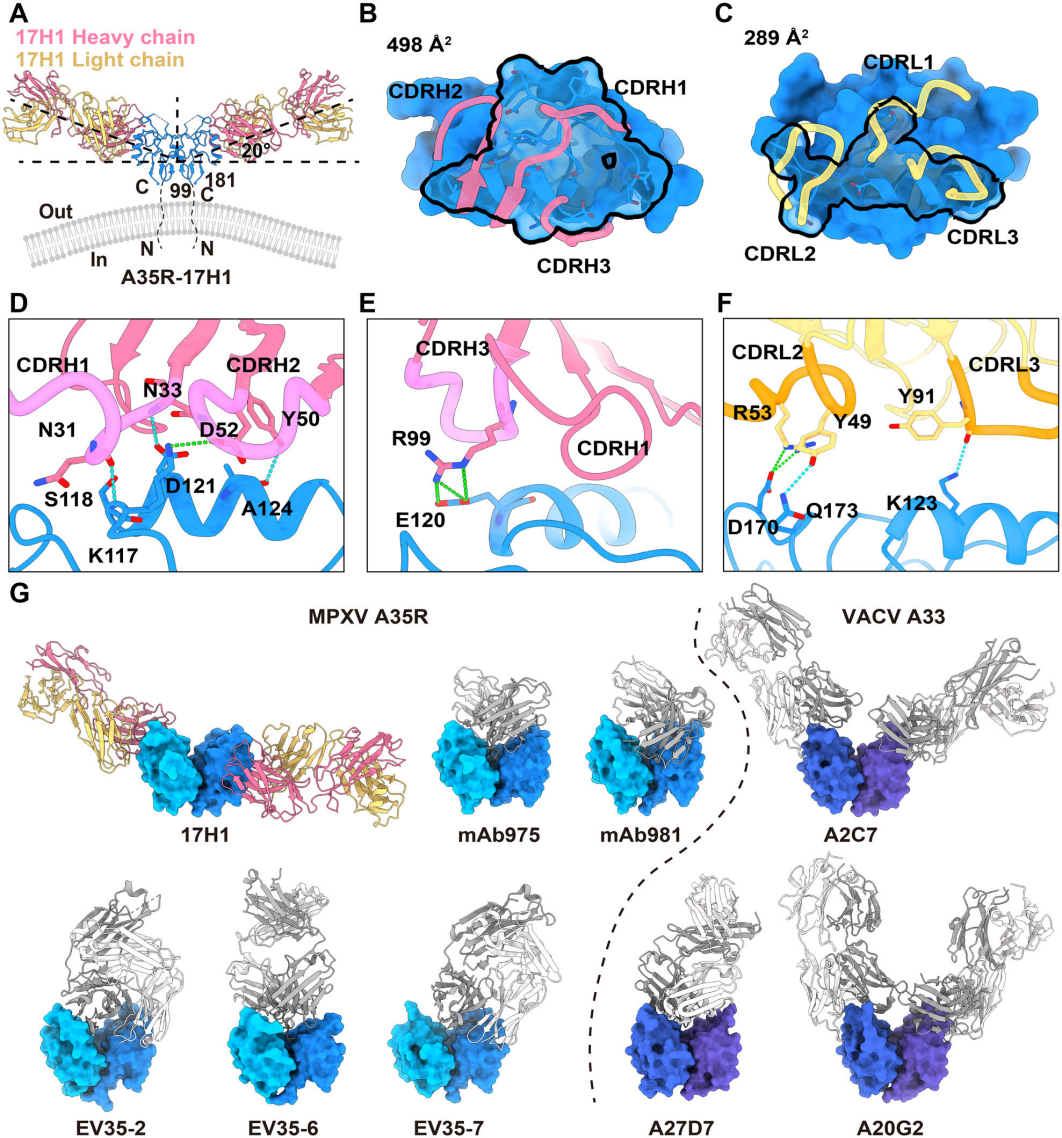
Interface analysis between 17H1 with MPXV A35R. (A) Overall structure of the MPXV A35R in complex with 17H1 Fab. (B and C) Surface area buried upon binding by the heavy (B) and light (C) chains of 17H1. (D–F) Detailed molecular interactions at the A35R‐17H1 interface, including hydrogen bonds and salt bridges formed by heavy chain CDR loops (D and E) and light chain CDR loops (F). Hydrogen bonds and salt bridges are marked by cyan and green dashed lines. Side chains involved in the interaction are labeled and shown as sticks. (G) Binding modes of MPXV A35R and VACV A33 in complex with Fab are shown. The A35R dimer is colored in light cyan and light blue, whereas the A33 dimer is colored in deep blue and deep purple. A35R and A33 are displayed in surface representation. Fab fragments are shown in ribbon representation, with the heavy and light chains colored in dark gray and snow, respectively.

Structural analysis showed that 17H1 recognizes epitopes located on the distal ends of the dimer. The Fabs engage A35R at a relative angle of approximately 20° (Figure 6A). Interface analysis revealed that binding is primarily mediated by the heavy chain of 17H1, which buries ∼498 Å^2^ of surface area (Figure 6B), compared to ∼289 Å^2^ for the light chain (Figure 6C). The interaction is stabilized by an extensive network of hydrogen bonds, salt bridges, and hydrophobic contacts. In the heavy chain of 17H1, CDRH1 (complementarity‐determining region 1 of heavy chain) contributes two hydrogen bond pairs (N31‐S118 and N33‐D121), while CDRH2 forms additional interactions with A35R (D52‐K117 and Y50‐A124) (Figure 6D, Table S5). A key interaction is mediated by R99 in CDRH3, which forms three salt bridges with E120 on A35R, identifying E120 as a critical epitope residue (Figure 6E, Table S5). In the light chain of 17H1, CDRL2 (complementarity‐determining region 2 of light chain) and CDRL3 contribute further stabilization via two hydrogen bond pairs (Y49‐Q173 and Y91‐K123) and two salt bridges (R53‐D170) (Figure 6F, Table S6). These interactions collectively account for the high binding affinity of 17H1 to A35R, and they suggest that 17H1 is conformationally dependent, unlike the previously reported isolated antibodies against the A35R, which target a linear epitope [20]. Compared to previously reported antibodies targeting A35R and the homologous A33 protein in VACV, including antibodies mAb975, mAb981, EV35‐2, EV35‐6, and EV35‐7 against A35R [26, 27], A2C7, A20G2, and A27D7 against A33 [18], 17H1 binds with the lowest flatness angle observed (Figure 6G), suggesting a unique mode of antigen recognition, which may contribute to its potent neutralizing ability to MPXV. Furthermore, sequence alignment showed that A35R maintains a high level of conservation among the four representative MPXV clades (including the clades Ib, Ia, IIb, and IIa), with nearly complete identity and greater than 98% homology to corresponding A35R proteins from diverse MPXV variants (Figure S5). It indicates that 17H1 may possess broad binding potential against currently prevalent MPXV clades Ib and IIb.

## 3 Discussion

Mpox continues to spread globally, particularly with sustained human‐to‐human transmission of the pandemic Clades Ib and IIb, posing a serious challenge to public health. Although historical smallpox vaccines can provide cross‐protection, their supply is limited, and adverse reactions remain a concern in susceptible populations. Furthermore, severe clinical manifestations and complications have been observed in immunocompromised individuals, highlighting the limitations of existing treatment methods [28, 29]. Therefore, it is urgent to develop novel intervention strategies to combat the mpox. While extensive antibody researches have been conducted on the surface proteins of intracellular mature virions (IMV), studies on the extracellular enveloped virions (EEV) unique to monkeypox virus (MPXV) remain incomplete. In our study, we focus on the MPXV A35R, a key immunogen on the EEV surface, aiming to develop effective therapeutic antibodies against MPXV.

To rapidly identify high‐performing monoclonal antibodies and address the poor immunogenicity associated with adjuvant‐free and aggregation‐limited recombinant proteins, we employed an A35R‐Fc mRNA‐LNP immunization regimen. Unlike traditional methods that rely on hybridoma fusion or mining convalescent sera, which may require a complex process or be limited by clinical sample requirements, our strategy leverages the potent immunogenicity of mRNA‐LNP and the rapid, optofluidic system‐based Beacon high‐throughput single‐cell sorting method. Finally, we successfully obtained five monoclonal antibodies, among which 17H1 outperformed others with picomolar affinity. Furthermore, in vivo experiments revealed that the 17H1 monoclonal antibody exhibits potent preventative and therapeutic potential against MPXV, significantly preventing weight loss and effectively reducing viral load in the lungs. Previous studies have shown low survival rates in mice treated with monoclonal antibodies targeting only a single viral form, suggesting the need for combined use of antibodies against both IMV and EEV forms to achieve synergistic and complete protection [30, 31, 32, 33]. However, recent research has made significant progress, demonstrating that single anti‐A35 targeting antibodies such as mAb 975, mAb 981, or EV35‐6 and EV35‐7 can protect mice from lethal MPXV infection [26, 27]. Our results also indicate that a single monoclonal antibody targeting the A35R protein is sufficient to halt disease progression and provide complete protection from death, but the protective mechanism requires further investigation.

Previous studies have confirmed that the neutralizing effect of EEV mAbs is complement‐dependent [15, 34, 35, 36]. Our results also show that the neutralizing activity of the A35‐targeting antibody against MPXV is significantly enhanced in the presence of complement. Interestingly, compared to its potent neutralizing activity against MPXV, 17H1 exhibits relatively weak neutralizing activity against VACV (results not shown). This difference may be attributed to the differences in key amino acid sites between the A35R antigen on MPXV‐EEV and the A33R antigen on VACV‐EEV [22], but the specific mechanism requires further investigation. Furthermore, sequence alignment shows that A35R is highly conserved across the four clades of MPXV (homology exceeding 98%), suggesting that 17H1 may have broad cross‐neutralizing activity against these variants, underscoring its potential as a candidate for preventing MPXV infection.

Extensive studies have been carried out on the functional role of A35R homologs (such as VACV A33), but detailed structural information regarding antibody‐antigen interfaces has been limited. In the study, we have resolved the high‐resolution structure of the MPXV A35R ectodomain in complex with the 17H1 Fab fragment, revealing the molecular basis of its potent neutralization. The structure highlights that 17H1 recognizes a unique conformational epitope located at the distal ends of the A35R dimer, engaging the antigen with a distinctively low flatness angle of approximately 20°. This mode of binding differs significantly from previously or newly reported antibodies that target the central “groove” of the dimer [26, 27], much like a pry bar. Structural analysis identified a critical salt bridge network (R118‐E120) and extensive hydrophobic contacts that stabilize this interaction. Furthermore, we observed that residues 57–98 of the A35R protein are flexible, which may explain the difficulties encountered in previous attempts to determine the full‐length protein structure. This suggests that the 17H1 antibody targets a structurally constrained and functionally critical region of the A35 protein, which may contribute to its higher affinity and ability to effectively prevent viral escape.

Our study has some limitations. Although we evaluated the protective efficacy of the 17H1 antibody against MPXV in BALB/c mice, future studies should test its effectiveness against different MPXV strains in other susceptible mouse models (CAST/EiJ or nude mice) [37, 38] or even non‐human primate models. Furthermore, the complement mechanism has been demonstrated to be involved in neutralizing orthopoxviruses by EEV antibodies in both humans [36] and mice [15, 34, 35]. However, other Fc‐mediated pathways may also contribute to the 17H1 antiviral efficacy, which requires further confirmation.

In summary, we used A35R‐Fc mRNA‐LNP immunization and the Beacon high‐throughput screening platform to produce five antibodies targeting the A35R protein. Among these, 17H1 exhibited potent MPXV neutralizing activity. Structural analysis elucidated its binding affinity and novel distal epitope information, suggesting that 17H1 is a promising candidate for the prevention and treatment of MPXV. Further investigation on its neutralization mechanism will also provide essential insights for developing novel immunotherapeutic strategies against MPXV or its variants.

## 4 Materials and Methods

### 4.1 Animals and Ethics Statement

Female BALB/c mice (6–8 weeks old) were purchased from Guangdong GemPharmatech and maintained under specific pathogen‐free (SPF) conditions at Shenzhen Third People's Hospital. All animal experiments were conducted in accordance with the Guide for the Care and Use of Laboratory Animals and were approved by the Ethics Committee of Shenzhen Third People's Hospital (approval number: 2024‐007). The study was conducted in accordance with the principles of the 3Rs and reported in compliance with the ARRIVE 2.0 guidelines. All MPXV infection experiments were performed in an Animal Biosafety Level 3 (ABSL‐3) facility in accordance with institutional biosafety and environmental health regulations. Euthanasia procedures were performed in accordance with institutional guidelines for animal care and use.

### 4.2 Cells and Virus

Vero‐E6 (ATCC, CRL‐1586) and HEK293T (ATCC, CRL‐3519) cells were cultured in Dulbecco's modified Eagle medium (DMEM, Thermo Fisher, USA), supplemented with 10% fetal bovine serum and 1% penicillin–streptomycin. Expi293F suspension cells (Thermo Fisher, USA, A14527) were maintained in serum‐free Expi293 Expression Medium (Thermo Fisher, USA, A1435102), and cultured at 37°C, in 5% CO_2_ with shaking at 120 rpm. All cell lines were routinely tested and confirmed to be free of mycoplasma contamination. Human cell lines, including HEK293T and Expi293F, were authenticated by STR profiling. The MPXV strain (GISAID accession ID EPI_ISL_18213374) was isolated from the first mpox case in Shenzhen, China.

### 4.3 Antigen‐specific Plasma B Cells Sorting, Antibody Cloning, Sequencing, and Production

The plasma B cells used in the Beacon high‐throughput screening system were derived from the mice immunized with the A35R‐Fc mRNA‐LNP vaccine (MPXV A35R, accession no. MT350282.1); the mRNA‐LNP formulations were prepared as previously described [39]. In brief, the vaccination was administered intramuscularly with 5 µg A35R‐Fc mRNA‐LNP using a prime‐boost scheme, in a 3‐week interval. Serum A35R‐specific antibody titers were measured 10 days following the booster injection. Next, we separated the spleen cells 7 days after the second immunization.

Spleens were collected and dissociated into single‐cell suspensions in RPMI. Total B cells were purified by magnetic negative selection (MojoSort Mouse Pan B Cell Isolation Kit; BioLegend, 480051) to remove non‐B cells. Antibody‐secreting cells (ASCs) were then enriched by magnetic positive selection of CD138+ cells (EasySep Mouse CD138+ Kit; Stem Cell, 18957) following the manufacturers’ instructions.

Isolated plasma B cells were loaded onto the Beacon platform (Berkeley Lights) and dispensed as single cells into individual nanopens. On‐chip ASC screening was carried out in Cell Analysis Suite using the standard assay script. Antigen‐coated assay beads (pre‐prepared with biotinylated A35R) were combined with FITC‐labeled goat anti‐mouse IgG (H+L) (Invitrogen, 31569). To detect antigen‐specific ASCs, the full chip (22 fields of view) was imaged in each fluorescence channel every 5 min for 30 min. Positive nanopens were then confirmed by the typical fluorescent “bloom” at the bead‐nanopen interface.

Single ASCs were exported into 96‐well plates containing TCL lysis buffer under mineral oil. RNA was purified with Agencourt RNAclean XP beads (Beckman Coulter, A63987) and reverse‐transcribed with random primers. Antibody variable regions were amplified by two rounds of nested PCR using the mouse Ig primer set (MilliporeSigma, 69831).

The amplifying mouse VH and VL sequences were cloned into the AbVec2.0‐IGHG1 (Addgene_80795) and AbVec1.1‐IGKC (Addgene_80796) mammalian expression vectors, which contained the human CH and CL constant sequences, respectively. The variable regions of mAbs were analyzed using Sanger sequencing with the universal SP6 primer, then determined using IgBLAST (http://www.ncbi.nlm.nih.gov/igblast) and IMGT (https://www.imgt.org/).

The heavy chain and light chain expression plasmids were transiently cotransfected into HEK293F cells at a mass ratio of 1:1.5. The supernatant was harvested 5 days later, and the mAbs were purified using protein A beads.

### 4.4 Flow Cytometry Analysis of the mAbs Binding to MPXV A35R

HEK293 T cells transfected with the MPXV‐A35R (full‐length) expression vectors. Cells were incubated with the five mAbs or an A35R‐specific antibody (Antibodysystem, RVV13102; purchased from AtaGenix Laboratories Co., Ltd., Wuhan, PR China), then stained with fluorescein isothiocyanate (FITC)‐labeled secondary antibody (Proteintech). After washing, the cells were measured on a BD FACSymphony A3 instrument.

### 4.5 Enzyme Linked Immunosorbent Assay (ELISA)

96‐well plates were coated with 100 ng/well of recombinant A35R protein (Novoprotein, Shanghai, China, DRA209) at 4°C overnight. Then, plates were blocked with 5% skim milk for 2 h at room temperature. Diluted mAbs were added into wells, then incubated at room temperature for 2 h. Plates were washed five times, HRP‐conjugated goat anti‐human IgG (H + L) (Proteintech, Wuhan, China, SA00001‐17) (1:5000) was added for 1 h at room temperature. Finally, tetramethylbenzidine was added and incubated for 5 min at room temperature in the dark. The reaction was stopped with 2 M H_2_SO_4_, and the absorbance at 450 nm was measured on a Synergy microplate reader (Bio‐Tek). EC_50_ values for the tested mAbs were calculated in GraphPad Prism 9.4 using a four‐parameter logistic (variable‐slope) nonlinear regression model (log[agonist] vs. response).

### 4.6 Surface Plasmon Resonance (SPR)

Binding assays of mAbs to the A35 protein (Novoprotein, Shanghai, China; DRA209) and competition between 17H1 and A27D7 were assessed by SPR on a Biacore 8K (GE Healthcare, UK). A CM5 sensor chip (Cytiva; 29149604) was used, with one flow cell amine‐coupled to A35R in 10 mM sodium acetate (pH 5.0) to around 200 RU, and the reference flow cell left blank and blocked. Experiments were run in HBS‐EP (Cytiva; BR100669) at 30 µL/min. For binding kinetics, serially diluted antibodies were injected for 60 s, and data were fit with a 1:1 interaction model in Biacore Evaluation software (GE, v3.0.12.15655). Measurements were performed in duplicate, and mean affinity values ± SD were reported. For competition assays, a saturation concentration of antibody (500 nM) was injected onto the A35 protein coated chip 120s until binding steady state was reached, followed immediately by injection of another antibody (500 nM) for 120 s. Competition status was determined by comparing RU values with and without prior occupancy by the primary antibody.

### 4.7 Focus Reduction Neutralization Test (FRNT)

Focus reduction neutralization tests (FRNTs) were used to assess mAb neutralizing activity. mAbs were 3‐fold serially diluted from 10 µg/mL, mixed with 200 focus‐forming units (FFUs) of MPXV in 96‐well plates, and incubated at 37°C for 1 h with 5% rabbit complement (Cedarlane, CL3441‑S100‑R). The mixtures were then added to Vero E6 monolayers (1.5 × 10^4^ cells/well) and incubated at 37°C for over 18 h. Cells were fixed with 4% paraformaldehyde for 60 min, permeabilized with BD Perm/Wash buffer containing 0.2% Triton X‑100 for 15 min, and stained with HRP‑conjugated anti‐vaccinia polyclonal antibody (Invitrogen, PA1‐73192) for 2 h at room temperature. Foci were developed with KPL TrueBlue peroxidase substrate (SeraCare, 5510‐0030) and counted using an EliSpot reader. IC_50_ values were calculated in GraphPad Prism 9.4 using a four‐parameter logistic (variable‐slope) model (log[inhibitor] vs. response). All MPXV FRNTs were performed in a BSL‐3 facility.

### 4.8 Animal Experiments

The prophylactic and therapeutic efficacy of the monoclonal antibody 17H1 was evaluated in vivo using 6‐ to 8‐week‐old BALB/c mice in a biosafety level 3 (BSL‐3) facility. Mice received either 20 µg of 17H1 (1 mg/kg) or 100 µg of 17H1 (5 mg/kg) intraperitoneally, either 24 h before or after challenge with MPXV (1 × 10^6^ FFU dose/mouse, 50 µL) intranasally. Mice treated with an equivalent quality of control IgG and challenged with a comparable dose of the MPXV served as negative controls. The body weight and survival rate were monitored daily. Five days post‐infection, three mice in each group were euthanized randomly. Then, half of the lung tissues were collected to quantify viral loads through TaqMan Real‐Time System (ABI QuantStudio), while the other half was fixed with 4% paraformaldehyde (PFA) and stained with hematoxylin and eosin to analyze the tissue's pathological morphology. A TaqMan probe (5‘‐FAM‐CATCAGAATCTGTAGGCCGT‐BHQ1‐3’) and the primer sequences for qPCR were as follows:

F3L‐F (5’‐CTCATTGATTTTTCGCGGGATA);

F3L‐R (5’‐GACGATACTCCTCCTCGTTGGT);

All samples were run in triplicate. The results analyses were performed using GraphPad Prism 9.4.

### 4.9 Cryo‐EM Sample Preparation

To generate Fab fragments of 17H1, monoclonal antibodies were digested with papain at a 1:1000 (w/w) ratio in 20 mM phosphate buffer (pH 7.0) containing 50 mM EDTA and 20 mM L‐cysteine. The reaction was carried out at 37°C for 12 h. Fab fragments were purified using Protein A resin (GenScript, Nanjing, China, L00210), which selectively removed the Fc portion. To assemble the immune complex, purified A35R and 17H1 Fab fragments were mixed at a molar ratio of 1:1.5. The mixture was subjected to Size‐exclusion chromatography to purify the complex through Superdex 200 Increase 10/300 column (GE Healthcare, USA, 28990944). The final sample was concentrated to approximately 0.4 mg/mL in preparation for cryo‐EM analysis. Then, 3 µL of the purified sample was applied to glow‐discharged Quantifoil R1.2/1.3 300 mesh grids coated with 200 nm continuous carbon. Grids were blotted and plunge‐frozen in liquid ethane using a Vitrobot Mark IV (Thermo Fisher Scientific) at 4°C and 100% humidity. The vitrified grids were then transferred and stored in liquid nitrogen for further imaging and analysis.

### 4.10 Data Collection and Processing

Cryo‐EM data were collected using a 200 kV Glacios 2 Cryo‐TEM (Thermo Fisher Scientific) equipped with a Falcon 4i direct electron detector camera. Images were recorded at a nominal magnification of 120,000×, corresponding to a calibrated pixel size of 1.2 Å. Each movie was captured over a 6‐second exposure, at a dose rate of 5.79 e^−^/pixel/s, yielding a total electron dose of ∼50 e^−^/Å^2^. Image processing was performed in cryoSPARC [40]. Movies were first subjected to motion correction. Defocus values and contrast transfer function (CTF) parameters were estimated using CTFFIND4 [41]. Initial particle picking was performed using Blob Picker, resulting in 1,660,603 particles. These particles were extracted with a box size of 300 pixels and subjected to 2D classification. Well‐defined classes were selected for ab initio reconstruction followed by heterogeneous refinement. From this, a subset of 157,067 particles exhibiting high‐quality features was chosen for non‐uniform refinement, yielding a density map at 4.40 Å resolution. To improve particle quality, template‐based particle picking was performed using a reference map generated from the initial reconstruction. This yielded 2,244,788 particles, which were subjected to 2D classification and heterogeneous refinement. The best particles from this round were merged with the previous high‐quality set, with duplicates removed. Final non‐uniform refinement of the merged dataset produced a reconstruction at 3.9 Å resolution. To further enhance map quality, local motion correction was applied using reference motion correction in cryoSPARC. These particles were subjected to non‐uniform refinement with C2 symmetry and produced a 3.71 Å map. A focused mask encompassing A35R and the variable domains of 17H1 was then applied for local refinement, resulting in a cryo‐EM map at 3.63 Å resolution. For further improving the quality of the cryo‐EM map, AR‐Decon [42], and EMReady [43] were used to generate the final map. All resolution estimates were based on the gold‐standard Fourier shell correlation (FSC) criterion at 0.143 [44]. Local resolution variations were visualized using ResMap [45].

### 4.11 Model Building and Refinement

The initial model of the A35R–17H1 was generated through AlphaFold 3 [46]. The model was fitted into the cryo‐EM density map using ChimeraX [47], followed by iterative manual adjustments in COOT [48] and Isolde to improve model‐map agreement. Real‐space refinement was performed using Phenix [49]. Regions lacking interpretable density were excluded from the model. The final model was validated using MolProbity [50]. All structural figures were prepared using ChimeraX. PISA server (https://www.ebi.ac.uk/pdbe/pisa/) and CCP4 [51] were used to analyze the interface interaction between A35R and 17H1.

### 4.12 Statistical Analysis

All data are presented as mean ± standard deviation (SD). Statistical analyses were performed using GraphPad Prism 9.4 (San Diego, CA, USA). One‐way ANOVA followed by Tukey's multiple‐comparisons test was used for comparisons among multiple groups. Survival curves were analyzed using the Kaplan–Meier method and compared using the log‐rank test. If the *p* value is less than 0.05, it is considered statistically significant.

## Author Contributions

S. B., S. S., and M. H. conceived and wrote the manuscript. X. W. and S. B. sorted B cells and cloned antibodies. S. B., Y. L., and Z. F. conducted the mice sample collection and performed sample measurements. Y. X. performed the structural studies. F. W. and F. W. reviewed the manuscript. H. L., J. Q., and M. H. guided the study and revised the manuscript. All authors have read and approved the final manuscript.

## Funding

This work was supported by the National Key Research and Development Program of China (Grant No.: 2023YFC2306400), the China Postdoctoral Science Foundation (Grant No.: 2025M771402), the National Natural Science Foundation of Shenzhen (Grant Nos.: JCYJ20250604143828037 and JCYJ20240813101859012), the Shenzhen Medical Research Fund (Grant No.: E24010010 and E24010014), the Special Funds for Strategic Emerging Industry of Shenzhen (Grant No.:F‐2022‐Z99‐502266), the Guangdong Medical Science and Technology Research Fund Project (Grant No.: A2023455) and the Research Funds from Hangzhou Institute for Advanced Study (Grant No.: 2024HIAS‐Y011).

## Conflicts of Interest

H.L. and S.B. are inventors on a pending Chinese patent application (Application No. CN202511661440.6; applicant: Shenzhen Third People's Hospital) related to the antibody sequences, screening methodologies, and protective efficacy of the therapeutic antibody and mRNA‐LNP formulation described in Figures 1, –4. The remaining authors declare no conflicts of interest.

## Ethics Statement

Animal studies were approved by the Ethics Committee of Shenzhen Third People's Hospital (approval number: 2024‐007). Euthanasia procedures strictly complied with ARRIVE reporting guidelines.

## Supporting information




**Figure S1**: Expression and purification results of all five mAbs
**Figure S2**: SPR‐based epitope competition assay to assess the binding interference between 17H1 and A27D7
**Figure S3**: Cryo‐EM sample preparation and data processing of A35R‐17H1 complex
**Figure S4**: Cryo‐EM density map of A35R‐17H1 complex structure
**Figure S5**: Conservative analysis of MPXV A35R homologous protein among orthopoxvirus
**Table S1**: Gene analysis of five MPXV‐A35R mAbs
**Table S2**: CDR sequences of five A35R protein‐specific antibodies
**Table S3**: Binding affinities of the 5 specific antibodies to A35R protein, measured by SPR
**Table S4**: Cryo‐EM data collection, refinement and validation statistics
**Table S5**: Interaction contacts between the heavy chain in 17H1 and A35R
**Table S6**: Interaction contacts between the light chain in 17H1 and A35R

## Data Availability

The cryo‐EM map and the coordinates of the A35R complexed with 17H1 have been deposited to the Electron Microscopy Data Bank (EMDB) and Protein Data Bank (PDB) with accession codes EMD‐80925, EMD‐80928 (local refinement), and 26WC (local refinement), respectively. Other data supporting the findings are included in the paper and/or the Supporting Information.
