# Supplementary material for: Discovery and Structural Characterization of a Highly Protective Neutralizing Antibody Targeting the Mpox Virus A35R Protein
Source: MedComm (2020). 2026 Jun 23;7(7):e70804. doi: 10.1002/mco2.70804 (PMC13291558; doi:10.1002/mco2.70804)
Supplement: Supplementary file 1 — Figure S1: Expression and purification results of all five mAbs Figure S2: SPR‐based epitope competition assay to assess the binding interference between 17H1 and A27D7 Figure S3: Cryo‐EM sample preparation and data processing of A35R‐17H1 complex Figure S4: Cryo‐EM density map of A35R‐17H1 complex structure Figure S5: Conservative analysis of MPXV A35R homologous protein among orthopoxvirus Table S1: Gene analysis of five MPXV‐A35R mAbs Table S2: CDR sequences of five A35R protein‐specific antibodies Table S3: Binding affinities of the 5 specific antibodies to A35R protein, measured by SPR Table S4: Cryo‐EM data collection, refinement and validation statistics Table S5: Interaction contacts between the heavy chain in 17H1 and A35R Table S6: Interaction contacts between the light chain in 17H1 and A35R [file MCO2-7-e70804-s001.docx]

**Discovery and structural characterization of a highly protective neutralizing antibody targeting the mpox virus A35R protein**

Shimeng Bai^1#^, Shuo Song^4,1#^, Xin Wang^1#^, Yuxin Xiao^2#^, Yun Long^1^, Fenfang Wu^1^, Fuxiang Wang^1^, Zhongyi Fan^1^, Jianqing Xu^3,1^*, Maozhou He^2*^ and Hongzhou Lu^1*^

^1^ Biotherapy Clinical Research Center, Shenzhen Key Laboratory of Pathogen and Immunity, National Clinical Research Center for Infectious Disease, Shenzhen Third People's Hospital, The Second Affiliated Hospital, Southern University of Science and Technology, Shenzhen, 518112, P. R. China

^2^ School of Pharmaceutical Science and Technology, Hangzhou Institute for Advanced Study, University of Chinese Academy of Sciences, Hangzhou, 310024, P. R. China

^3^ Clinical Center of Biotherapy, Zhongshan Hospital & Institutes of Biomedical Sciences, Fudan University, Shanghai, 200032, P. R. China

^4^ Faculty of Life and Health Sciences, Shenzhen University of Advanced Technology, Shenzhen, 518107, P. R. China

*Corresponding authors: Jianqing Xu, xujianqing@fudan.edu.cn; Maozhou He, hemaozhou@ucas.ac.cn; Hongzhou Lu, luhongzhou@fudan.edu.cn.

Shimeng Bai, Shuo Song, Xin Wang, and Yuxin Xiao contribute equally to this work.

**Figure S1 Expression and purification results of all five mAbs**

The 55 kDa and 25 kDa bands on the gel represent the heavy chain (HC) and light chain (LC) of the antibody, as observed in the SDS-PAGE gel test.

**
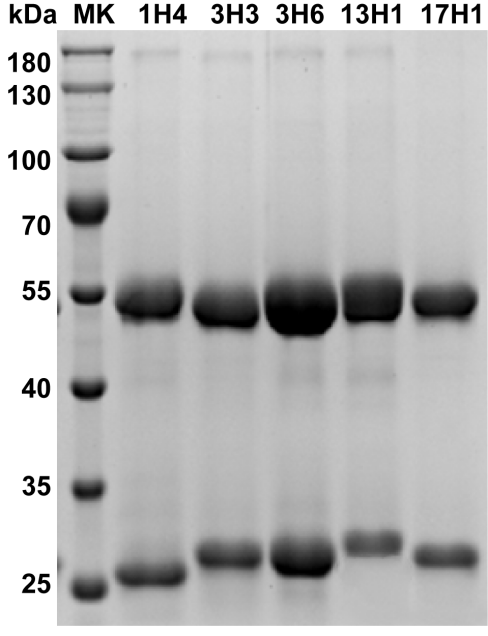
**

**Figure S2 SPR-based epitope competition assay to assess the binding interference between 17H1 and A27D7**

The immobilized antigen was pre-saturated with one antibody before introducing another antibody to the sensor surface. Individual binding events with and without prior occupancy by the primary antibody are color-coded differentially (A). Binding affinities of the A27D7 to A35R protein, measured by SPR (B). Each assay was performed twice.

**
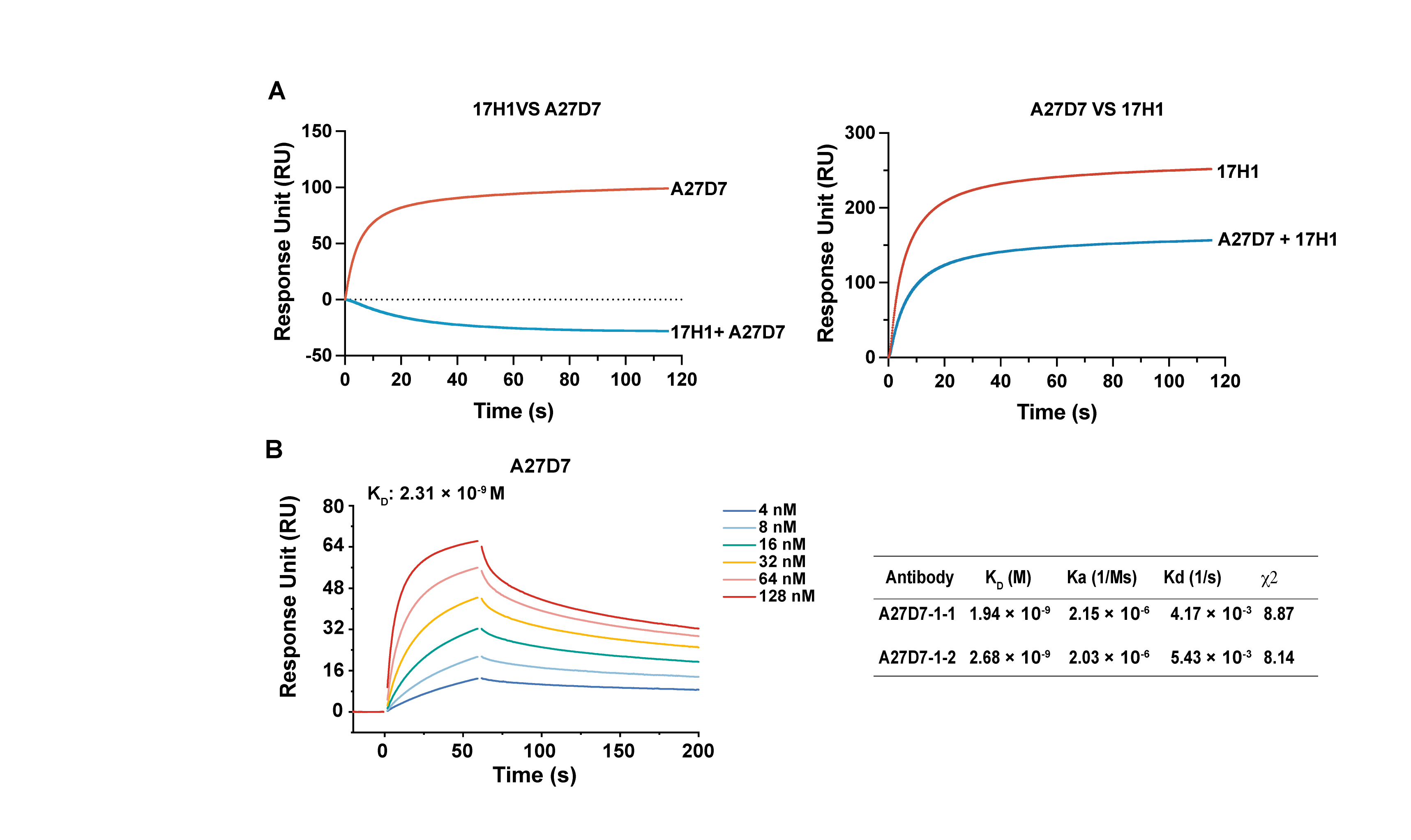
**

**Figure S3 Cryo-EM sample preparation and data processing of A35R-17H1 complex**

Gel-filtration analysis (A) and SDS-PAGE (B) of MPXV A35R in complex with 17H1 Fab. (C) Cryo-EM raw images of A35R–17H1 complex. (D) Data processing flowchart of A35R-17H1 complex.


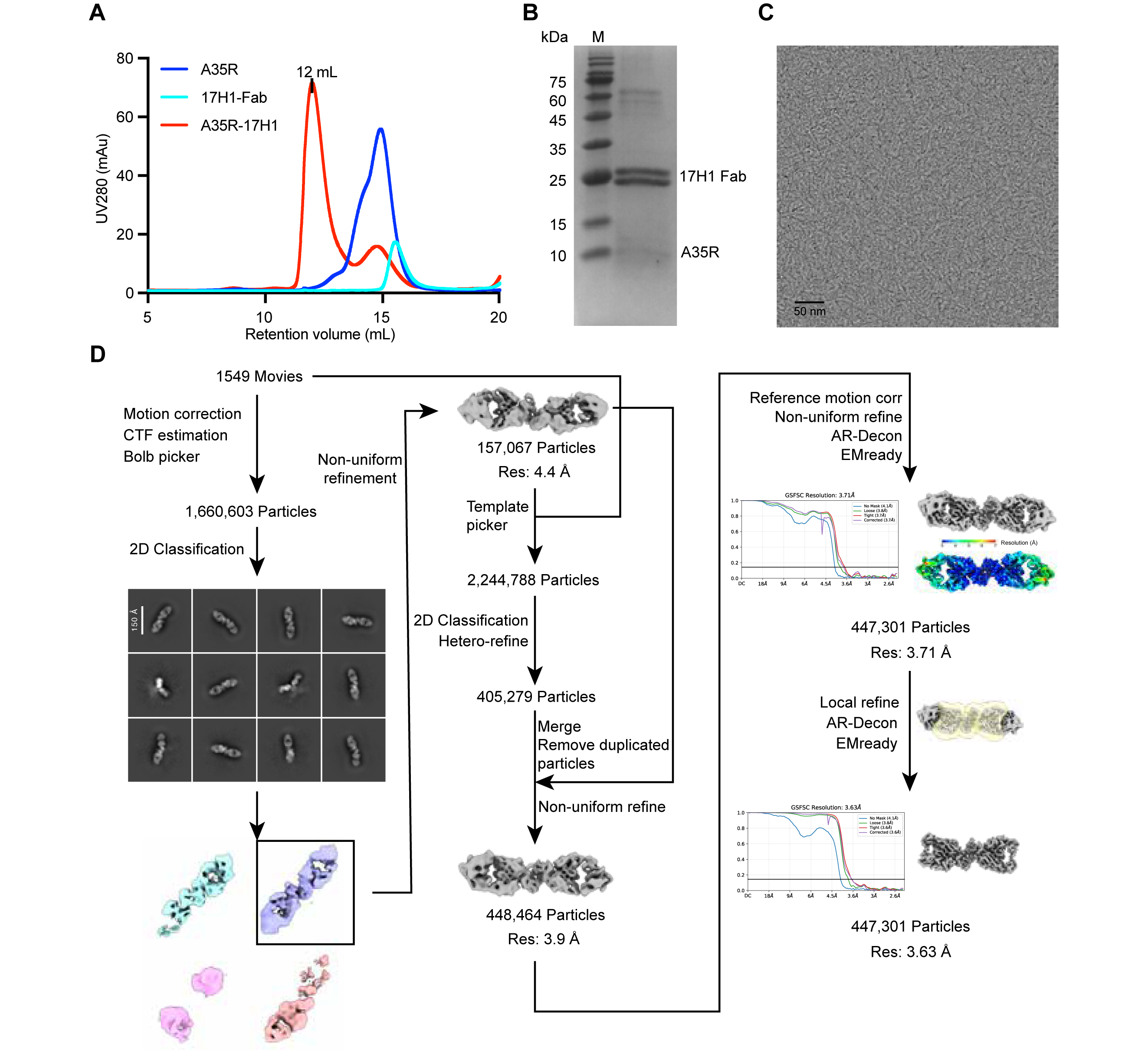


**Figure S4 Cryo-EM density map of A35R-17H1 complex structure**

Cryo-EM density maps and models in the interaction interface between A35R (A), 17H1 heavy chain (B) and light chain (C). The models are shown as sticks and colored according to chains.

**
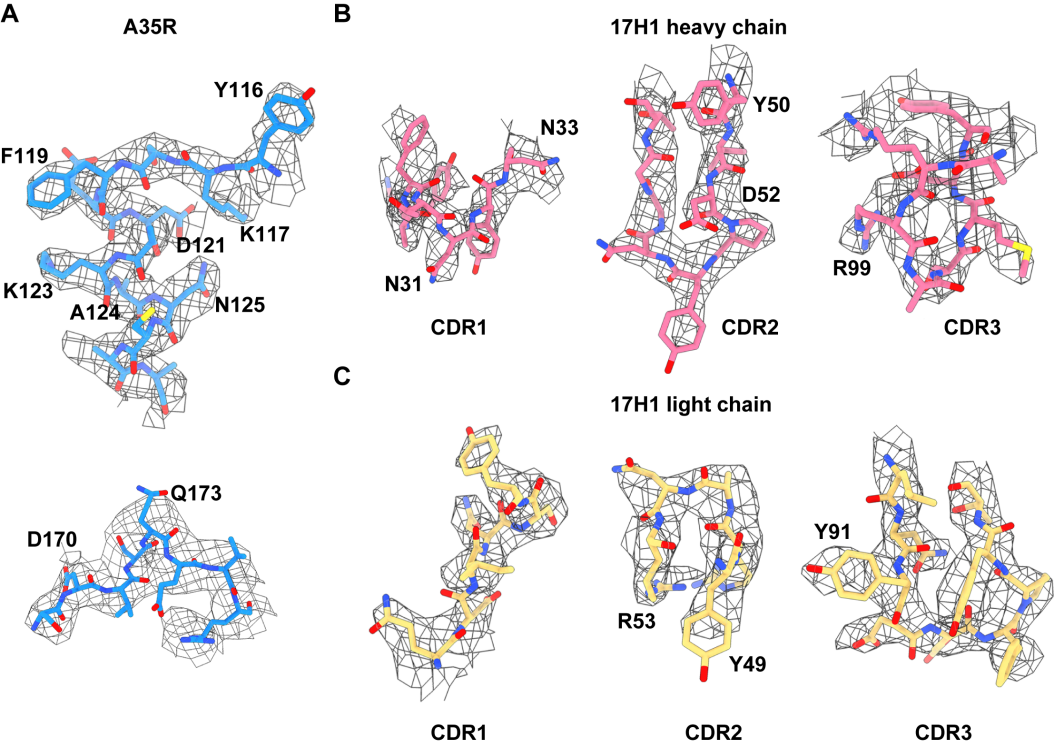
**

**Figure S5 Conservative analysis of MPXV A35R homologous protein among orthopoxvirus**

Sequence alignment (A) and identity (B) of MPXV A35R in different kinds of orthopoxvirus: MPXV_24MPX0198V strain,accession no. WZB45693, clade Ib; MPXV_Zaire-96-I-16 strain, accession no. NP_536572, clade Ia; MPXV_USA_2022_MA001 strain, accession no.URK20584, clade IIb; MPXV_W-Nigeria strain, accession no.AIE40758, clade IIa; VACV_Tiantan strain, accession no.AGJ91350; VACV_Western Reserve strain, accession no. YP_233038; VARV_India-1958 strain, accession no. WWS34586; CPXV_GRI-90 strain, accession no. CAD90701.

**
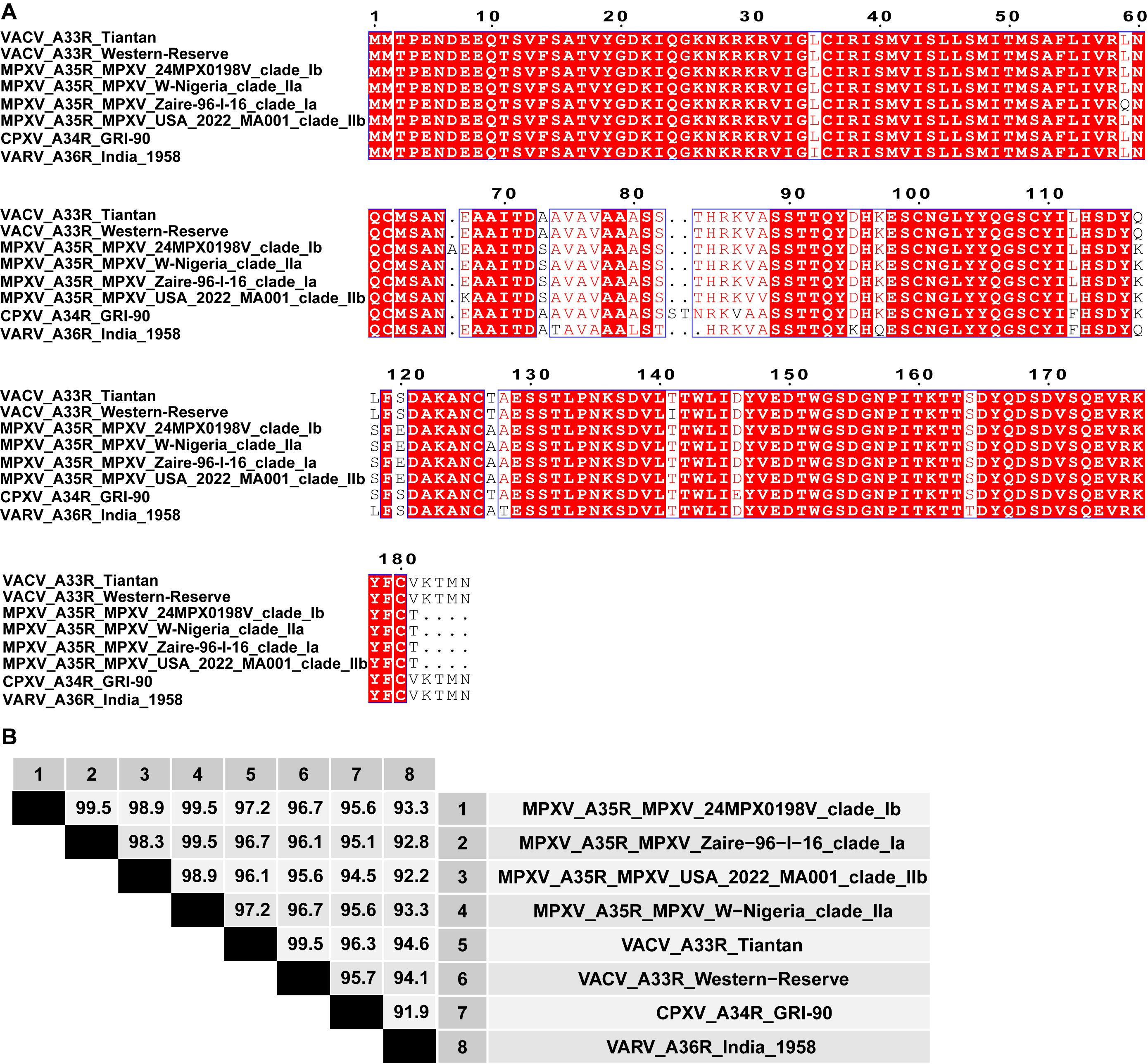
**

**Table S1 Gene analysis of five MPXV-A35R mAbs**

| **mAbs** | **Heavy  chain** | | | **Light chain** | | |
| --- | --- | --- | --- | --- | --- | --- |
|  | **IGHV** | **CDR3(aa）** | **SHM(%)** | **IGKV** | **CDR3(aa）** | **SHM(%)** |
| 1H4 | V1-85*02 | 19 | 3.50 | V6-14*01 | 9 | 2.10 |
| 3H3 | V1-18-28*01 | 7 | 3.80 | V14-111*01 | 9 | 1.10 |
| 3H6 | V1-7*02 | 11 | 4.40 | V14-111*01 | 9 | 1.10 |
| 13H1 | V1-18-28*01 | 9 | 2.40 | V3-10*01 | 9 | 0.30 |
| 17H1 | V1-18-28*01 | 8 | 2.70 | V14-111*01 | 9 | 1.40 |

**Table S2 CDR sequences of five A35R protein-specific antibodies**

| **mAb** | **Amino acid sequences of CDRs** |
| --- | --- |
| 1H4 | K-CDRs:QNVHTT......_LAS......_LQHWNYPLT |
|  | H-CDRs:GYTFTSYD......_IFPGDGTT......_ARAGPPRPIYGNYLAWFAY |
| 3H3 | K-CDRs:QDINSY......_RAN......_LQYDEFPYT |
|  | H-CDRs:GYAFTSYN......_IDPYNGNT......_ARLLRDF |
| 3H6 | K-CDRs:QDINSY......_RAN......_LQYDEFPYT |
|  | H-CDRs:GYTFTTYW......_INPSTGYT......_ARSRNGYGFAY |
| 13H1 | K-CDRs:ESVDSYGNSF......_LAS......_QQNNEDPWT |
|  | H-CDRs:GYAFTSYN......_IDPYNGGT......_ARDYWYFDV |
| 17H1 | K-CDRs:QDINSY......_RAN......_ LQYDEFPYT |
|  | H-CDRs:GYAFTNYN......_IDPYNGGT......_ARRASMDY |

**Table S3 Binding affinities of the 5 specific antibodies to A35R protein, measured by SPR**

| **Antibody** | **K_D_ (M)** | **Ka (1 / Ms)** | **Kd (1/s)** | **ꭓ2** |
| --- | --- | --- | --- | --- |
| 1H4-1 | 2.25 ×10 ^-10^ | 2.51 × 10^5^ | 5.65 × 10^-5^ | 0.98 |
| 1H4-2 | 1.88 × 10 ^-10^ | 1.83 × 10^6^ | 3.45 × 10 ^-4^ | 0.235 |
| 3H3-1 | 1.42 × 10 ^-9^ | 1.75 × 10^6^ | 2.48 × 10 ^-3^ | 17.3 |
| 3H3-2 | 1.77 ×10 ^-9^ | 1.06 × 10^6^ | 1.88 ×10 ^-3^ | 14.3 |
| 3H6-1 | 2.65 ×10 ^-9^ | 9.85 × 10^5^ | 2.61 × 10 ^-3^ | 44.6 |
| 3H6-2 | 2.28 × 10 ^-9^ | 9.43 × 10^5^ | 2.15 × 10 ^-3^ | 50.7 |
| 13H1-1 | 6.24 × 10 ^-10^ | 4.72 × 10^5^ | 2.94 × 10 ^-4^ | 1.17 |
| 13H1-2 | 2.13 × 10 ^-10^ | 5.21 × 10^5^ | 1.11 × 10^-4^ | 1.43 |
| 17H1-1 | 8.93 × 10 ^-11^ | 1.68 × 10^6^ | 1.50 ×10 ^-4^ | 3.55 |
| 17H1-2 | 9.12 × 10 ^-11^ | 1.93 × 10^6^ | 1.76 × 10 ^-4^ | 1.83 |

**Table S4 Cryo-EM data collection, refinement and validation statistics**

|  | **A35R-17H1** | **A35R-17H1**  **(local refinement)** |
| --- | --- | --- |
| Magnification | 120,000 | 120,000 |
| Voltage (kV) | 200 | 200 |
| Electron exposure (e^–^/Å^2^) | 50 | 50 |
| Defocus range (μm) | –1.0 ~ –2.0 | –1.0 ~ –2.0 |
| Pixel size (Å) | 1.2 | 1.2 |
| Symmetry imposed | C2 | C2 |
| Initial particle images (no.) | 1,549 | 1,549 |
| Final particle images (no.) | 447,301 | 447,301 |
| Map resolution (Å) | 3.71 | 3.63 |
| FSC threshold | 0.143 | 0.143 |
| Map resolution range (Å) | 3.0–7.0 | 3.0–7.0 |
| Initial model used (PDB code) | Alphafold3 | Alphafold3 |
| Model resolution (Å) |  | 3.8 |
| FSC threshold |  | 0.5 |
| Map sharpening *B* factor (Å^2^) | –186 | –172 |
| Model composition |  |  |
| Non-hydrogen atoms |  | 4,770 |
| Protein residues |  | 610 |
| Lipid |  | 0 |
| *B* factors (Å^2^) |  |  |
| Protein |  | 69.46 |
| Lipid |  | / |
| R.m.s. deviations |  |  |
| Bond lengths (Å) |  | 0.003 |
| Bond angles (°) |  | 0.924 |
| Validation |  |  |
| MolProbity score |  | 1.52 |
| Clashscore |  | 4.31 |
| Poor rotamers (%) |  | 0.00 |
| Ramachandran plot |  |  |
| Favored (%) |  | 95.62 |
| Allowed (%) |  | 4.38 |
| Disallowed (%) |  | 0.00 |

**Table S5 Interaction contacts between the heavy chain in 17H1 and A35R**

| **A35R** | **Distance (Å)** | **Fab-17H1 (heavy chain)** | **CDR** |
| --- | --- | --- | --- |
| S114 [OG] | 3.99 | Y54 [OH] | CDR2 |
| D115 [CB] | 3.77 | Y54 [CG] | CDR2 |
| D115 [CB] | 3.90 | Y54 [CD1] | CDR2 |
| D115 [OD2] | 3.58 | Y54 [CB] | CDR2 |
| D115 [OD2] | 3.85 | Y54 [CG] | CDR2 |
| D115 [OD2] | 3.93 | Y54 [CD2] | CDR2 |
| D115 [OD2] | 3.98 | D52 [OD2] | CDR2 |
| Y116 [O] | 3.62 | T30 [O] | CDR1 |
| Y116 [O] | 3.34 | N31 [CA] | CDR1 |
| Y116 [O] | 3.84 | N31 [C] | CDR1 |
| Y116 [O] | 3.56 | N31 [OD1] | CDR1 |
| Y116 [O] | 3.57 | N31 [O] | CDR1 |
| Y116 [CB] | 3.41 | N31 [OD1] | CDR1 |
| K117 [CA] | 3.45 | N31 [O] | CDR1 |
| K117 [CD] | 3.71 | T30 [O] | CDR1 |
| K117 [CD] | 3.83 | N31 [C] | CDR1 |
| K117 [CD] | 3.21 | N31 [O] | CDR1 |
| K117 [CE] | 3.30 | T30 [O] | CDR1 |
| K117 [CE] | 3.30 | D52 [OD1] | CDR2 |
| K117 [NZ] | 2.96 | T30 [O] | CDR1 |
| K117 [NZ]^#^ | 3.00 | D52 [OD1] | CDR2 |
| K117 [NZ] | 3.82 | Y32 [C] | CDR1 |
| K117 [NZ] | 3.69 | N33 [N] | CDR1 |
| K117 [NZ] | 3.49 | P53 [CD] | CDR2 |
| S118 [N]^*^ | 3.85 | N31 [O] | CDR1 |
| S118 [CB] | 3.94 | Y32 [CE1] | CDR1 |
| S118 [OG] | 3.94 | Y32 [CE1] | CDR1 |
| S118 [OG] | 3.67 | Y32 [CD1] | CDR1 |
| S118 [OG] | 3.76 | R99 [CG] | CDR3 |
| S118 [OG] | 3.99 | R99 [CD] | CDR3 |
| S118 [OG] | 3.77 | R99 [NE] | CDR3 |
| E120 [CB] | 3.66 | A100 [CB] | CDR3 |
| E120 [CD] | 3.21 | R99 [NH2] | CDR3 |
| E120 [OE1] ^#^ | 3.58 | R99 [NE] | CDR3 |
| E120 [OE1] | 3.66 | R99 [CZ] | CDR3 |
| E120 [OE1] ^*, #^ | 2.86 | R99 [NH2] | CDR3 |
| E120 [OE2] ^#^ | 2.98 | R99 [NH2] | CDR3 |
| E120 [OE2] | 3.97 | E101 [OG] | CDR3 |
| D121 [CG] | 3.93 | N33 [N] | CDR1 |
| D121 [CG] | 3.45 | N33 [CG] | CDR1 |
| D121 [CG] | 3.84 | N33 [ND2] | CDR1 |
| D121 [CG] | 3.24 | N33 [OD1] | CDR1 |
| D121 [OD1] | 3.81 | N33 [CB] | CDR1 |
| D121 [OD1] | 3.01 | N33 [CG] | CDR1 |
| D121 [OD1] | 2.99 | N33 [ND2] | CDR1 |
| D121 [OD1] | 3.14 | N33 [OD1] | CDR1 |
| D121 [OD2] | 3.98 | N31 [O] | CDR1 |
| D121 [OD2] | 3.58 | Y32 [C] | CDR1 |
| D121 [OD2] ^*^ | 2.81 | N33 [N] | CDR1 |
| D121 [OD2] | 3.77 | N33 [CA] | CDR1 |
| D121 [OD2] | 3.57 | N33 [CB] | CDR1 |
| D121 [OD2] | 3.24 | N33 [CG] | CDR1 |
| D121 [OD2] | 3.43 | Y32 [CA] | CDR1 |
| D121 [OD2] | 2.93 | N33 [OD1] | CDR1 |
| A124 [O]^*^ | 3.37 | Y50 [OH] | CDR2 |
| A124 [CB] | 3.61 | Y35 [OH] | FR2 |
| N125 [OD1] | 3.97 | D52 [CG] | CDR2 |
| N125 [OD1] | 3.69 | D52 [OD2] | CDR2 |
| N125 [OD1] | 3.88 | Y50 [CE2] | CDR2 |
| N125 [ND2] | 4.00 | D52 [OD2] | CDR2 |
| A128 [CB] | 3.10 | Y50 [OH] | FR2 |
| V175 [CB] | 3.67 | N31 [ND2] | CDR1 |
| V175 [CG1] | 3.94 | N31 [CG] | CDR1 |
| V175 [CG1] | 3.43 | N31 [ND2] | CDR1 |
| V175 [CG1] | 3.90 | Y32 [CE1] | CDR1 |
| V175 [CG1] | 3.95 | Y32 [CZ] | CDR1 |
| V175 [CG1] | 3.51 | Y32 [OH] | CDR1 |
| V175 [CG2] | 3.36 | N31 [ND2] | CDR1 |

^*^ refers to the hydrogen bonds.

^#^ refers to the salt-bridge.

**Table S6 Interaction contacts between the light chain in 17H1 and A35R**

| **A35R** | **Distance (Å)** | **Fab-17H1 (light chain)** | **CDR** |
| --- | --- | --- | --- |
| F119 [CE2] | 3.52 | Y32 [OH] | CDR1 |
| F119 [CZ] | 3.40 | Y32 [OH] | CDR1 |
| K123 [CD] | 3.71 | Y32 [OH] | CDR1 |
| K123 [CE] | 3.80 | Y32 [CE2] | CDR1 |
| K123 [CE] | 3.67 | Y32 [CZ] | CDR1 |
| K123 [CE] | 3.70 | Y32 [OH] | CDR1 |
| K123 [NZ]^*^ | 3.21 | Y91 [O] | CDR3 |
| A124 [O] | 3.61 | F94 [CE2] | FR3 |
| A124 [O] | 3.67 | F94 [CZ] | FR3 |
| A124 [CB] | 3.39 | Y96 [OH] | CDR3 |
| A127 [C] | 3.97 | F94 [CE2] | FR3 |
| A127 [CB] | 3.63 | F94 [CE2] | FR3 |
| Q173 [NE2] ^*^ | 3.32 | Y49 [OH] | FR2 |
| D170 [OD2] ^*, #^ | 2.92 | R53 [NE] | CDR2 |
| D170 [OD2] | 3.33 | R53 [CZ] | CDR2 |
| D170 [OD2] ^#^ | 3.11 | R53 [NH2] | CDR2 |
| D170 [OD2] | 3.94 | R53 [CD] | CDR2 |

^*^ refers to the hydrogen bonds.

^#^ refers to the salt-bridge
